# The interplay of movement and spatiotemporal variation in transmission degrades pandemic control

**DOI:** 10.1073/pnas.2018286117

**Published:** 2020-11-10

**Authors:** Nicholas Kortessis, Margaret W. Simon, Michael Barfield, Gregory E. Glass, Burton H. Singer, Robert D. Holt

**Affiliations:** ^a^Department of Biology, University of Florida, Gainesville, FL 32611;; ^b^Department of Geography, University of Florida, Gainesville, FL 32611;; ^c^Emerging Pathogens Institute, University of Florida, Gainesville, FL 32610

**Keywords:** spatiotemporal variation, COVID-19, inflationary effect

## Abstract

Successful public health regimes for COVID-19 push below unity long-term regional *R*_*t*_ —the average number of secondary cases caused by an infectious individual. We use a susceptible-infectious-recovered (SIR) model for two coupled populations to make the conceptual point that asynchronous, variable local control, together with movement between populations, elevates long-term regional *R*_*t*_, and cumulative cases, and may even prevent disease eradication that is otherwise possible. For effective pandemic mitigation strategies, it is critical that models encompass both spatiotemporal heterogeneity in transmission and movement.

… we’re a large country that has outbreaks in different regions, different states, different cities, that have different dynamics, and different phases…

Anthony Fauci, quoted on CNN, 23 April 2020 (1)

To control COVID-19, public policy must drive average effective net reproduction number (*R*_*t*_) below unity, globally. Yet local governments craft policies based on local trends. In the United States, local controls wax and wane over time, often poorly coordinated across polities (e.g., states). New York may surge while Florida does not, but, later, this flips, generating repeated outbreaks varying among locations. Mathematical models are essential tools to monitor and control epidemics such as COVID-19 (2–6), but they can also sharpen intuition about emergent epidemiological phenomena. While spatial processes are increasingly incorporated into epidemiological theory (7), as is temporal variation in disease transmission (4, 5, 8), their combined effect is underappreciated. Yet spatiotemporal variation is pervasive in epidemics; COVID-19 is no exception. Data for the United States (e.g., https://rt.live/) suggest *R*_*t*_ has fluctuated in wave-like fashions, with peaks at different times in different states. We show that infectious individuals moving among populations with asynchronous temporal dynamics in transmission can permit disease persistence when extirpation would otherwise occur, based on local estimates of transmission: Global average *R*_*t*_ over time may exceed 1, despite time-averaged *R*_*t*_ < 1 locally, everywhere. Moreover, even when extirpation is unlikely, spatiotemporally heterogeneous transmission, coupled with movement, can accelerate epidemic spread.

We illustrate these generic features of pandemics using a susceptible-infectious-recovered (SIR) model (see *Materials and Methods*) that captures essential elements of more realistic models, and allows us to clarify the essential features responsible for such effects. We consider 1) effective local control, where local transmission dynamics imply eradication, were locations isolated; and 2) ineffective local control, where local transmission dynamics generate varying but sustained spread.

## Results

**Fig. 1.**
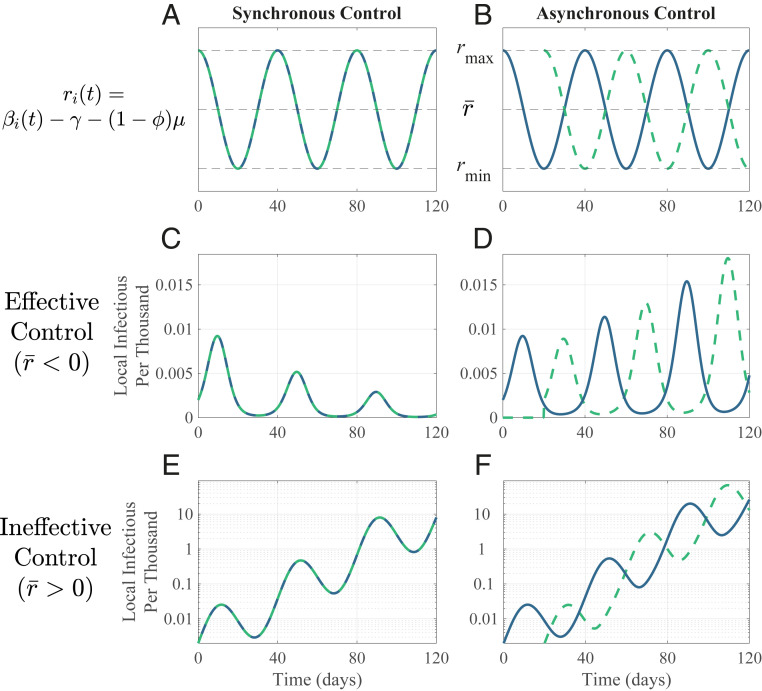
Movement among asynchronous populations enhances disease spread. Sinusoidal transmission dynamics alternate between increasing (*r*_max_ > 0; *R*_*t*_ > 1) and declining prevalence (*r*_min_ < 0; *R*_*t*_ < 1). Two populations (dashed; solid) exactly (*A*) in-phase (Ω = 0) and (*B*) out of phase (Ω = 1). Number of infectious individuals in each population over time with effective control for (*C*) synchronous and (*D*) asynchronous populations, and for ineffective control with (*E*) synchronous and (*F*) asynchronous populations (note log scale). Populations differ only in timing of transmission dynamics. Movement rate *m* = 0.005 d^−1^. For other parameters, see *Materials and Methods*.

Fig. 1 shows these two scenarios for local populations, either synchronous or asynchronous in their time-varying control efforts, with sinusoidal local transmission. Given effective local control (local *r̅* < 0; time-averaged *R*_*t*_ < 1), the disease cycles due to fluctuating transmission rates, but declines overall in isolated populations. The same holds for synchronized populations connected by movement (Fig. 1*C*). But, with poor coordination (asynchrony), the disease instead spreads (Fig. 1*D*). Merely changing the relative timing of local controls reverses global outcomes. Either synchronizing policies or cutting movement between local populations drives the disease extinct.

Given ineffective control (i.e., local *r̅* > 0; time-averaged *R*_*t*_ > 1), the disease cycles, with increasing peaks for synchronous populations (Fig. 1*E*). With asynchrony, the disease overall increases faster, with higher infection peaks (Fig. 1*F*): Movement and asynchrony jointly elevate global infections. In this example, early in the epidemic (i.e., *S*_*i*_ = *N*_*i*_), asynchrony increases the net change over the cycle from 0.073 d^−1^ to 0.099 d^−1^ (the average weekly increase goes from 67 to 100%); this boost in infectious spread is evident after a few cycles.

**Fig. 2.**
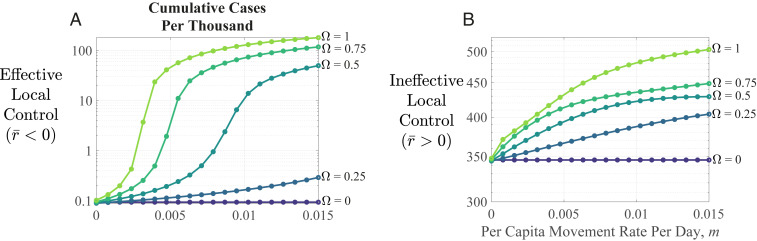
Long-term disease outcomes. Cumulative cases per thousand postpandemic increase with asynchrony (Ω), whether local control is (*A*) effective (note log scale) or (*B*) ineffective. Parameters are as in Fig. 1.

The populations in these examples are completely out of sync, but this is not required for asynchrony to hasten disease spread. Fig. 2 displays total cases after the pandemic has run its course, as a function of asynchrony (Ω) and movement (*m*); even modest asynchrony and movement boost cumulative cases.

## Discussion

Our goal here is to illustrate conceptually a qualitative effect: If 1) local transmission patterns vary through time and across space, and 2) some infectious individuals move between populations, overall control is hampered. Intuitively, why does this occur? Immigration of infectious individuals into a location during periods when *R*_*t*_ < 1 (a “sink” location, e.g., during lockdowns) maintains prevalence greater than expected, and those locations can then surge to greater prevalence when they switch to *R*_*t*_ > 1 (a “source,” when lockdowns ease). If different localities enter and leave lockdown at different times, they, in effect, take turns sourcing and receiving immigrants, fueling disease spread reciprocally. Prevalence increases with the time that populations differ in *R*_*t*_, explaining why prevalence increases with asynchrony. Longer cycle lengths (for given asynchrony) further elevate disease spread. Such effects are known in population ecology under the rubric of the “inflationary effect” (9, 10), emerging from spatiotemporal variation among populations coupled by movement—quintessential features of pandemics.

Many real-world complexities need consideration to apply this insight, in detail, to the COVID-19 pandemic (e.g., refs. 3, 5, 11). However, the emergent effect we identify—heightened global prevalence due to movement linking asynchronous locations—pertains to realistic scenarios and likely arises in realistic models of any pandemic. A recent study (12) is an example, concluding that failure to coordinate lifting of lockdowns across Europe could substantially increase community transmission. Our results suggest such effects should occur generically in many pandemics.

These findings highlight a need for integrated, holistic policy: Intensify mitigation locally, coordinate tactics among locations, and reduce movement. It is increasingly recognized that monitoring and controlling movement is essential for effective pandemic control (13). The impact of such actions is, however, contextual, because their dynamical effects are intertwined with the magnitude of asynchrony in local transmission across space. More-realistic, spatially structured epidemiological models including movement and asynchronous transmission—at scales from local to international—are essential to control this and future pandemics in the coupled metapopulations of humans and their pathogens.

## Materials and Methods

### SIR Dynamics.

Two populations (*i, j = A, B*, *i ≠ j*) each follow a time-varying SIR model,

$$\frac{dS_{i}}{dt}=-\frac{S_{i}I_{i}}{N_{i}}\beta_{i}\left(t\right)-mS_{i}+mS_{j}$$

$$\frac{dI_{i}}{dt}=\frac{S_{i}I_{i}}{N_{i}}\beta_{i}\left(t\right)-\gamma I_{i}-\left(1-\phi \right)\mu I_{i}-m\phi I_{i}+m\phi I_{j}$$

$$\frac{dR_{i}^{'}}{dt}=\gamma I_{i}-mR_{i}^{'}+mR_{j}^{'}.$$ [1]

*S*_*i*_, *I*_*i*_, and *R*_*i*_′ are local susceptible, infectious, and recovered individuals, (*N*_*i*_ = *S*_*i*_ + *I*_*i*_ + *R*_*i*_′ is local population size, assumed 5 million). *β*_*i*_(*t*), *γ*, *μ*, and *m* are transmission, recovery, disease mortality, and movement rates, respectively, and *ϕ* is fraction of infections without symptoms (symptomatic individuals are immobile). When COVID-19 is rare, *S*_*i*_ ≈ *N*_*i*_, and disease dynamics follow (*i* ≠ *j*),

$$\frac{dI_{i}}{dt}=r_{i}\left(t\right)I_{i}-m\phi I_{i}+m\phi I_{j},\;\;\;i=A,B,$$ [2]

where *r*_*i*_(*t*) = *β*_*i*_(*t*) – *γ* – (1 – *ϕ*)*μ* is local per capita rate of change in the infectious class without movement. *I*_*i*_ increases for *r*_*i*_(*t*) > 0 and declines for *r*_*i*_(*t*) < 0. We assume *r*_*i*_(*t*) varies sinusoidally around its average, *r̅*, with period *T* (assumed 40 d; Fig. 1), approximating waves of disease spread and decline due to shifting policy and behavior, with maximum and minimum values of *r*_max_ > 0 and *r*_min_ < 0. *I*_*i*_ asymptotically declines locally when *r̅* = (*r*_max_ + *r*_min_)/2 < 0 and spreads locally when *r̅* > 0. In one case, local conditions cause local decline (*r̅* < 0; “effective local control”); in another, they do not (*r̅* > 0, “ineffective local control”). Asynchrony Ω is the phase shift between the locations (= [1 – cos(2*πτ/T*)]/2), where *τ* is the time shift in days between populations’ *r*_*i*_. Completely in-phase *r*_*i*_(*t*) implies Ω = 0, and exactly out-of-phase *r*_*i*_(*t*) implies Ω = 1.

We used empirical estimates (range and references in parentheses) for COVID-19 parameters. For *r̅* < 0, *β* varies between 0.57 and 0.045 d^−1^ (0.09 to 1.12; refs. 3, 4) and *γ* = 0.32 d^−1^ (0.07 to 0.29; refs. 3, 4, 11). We approximated *ϕ* as the fraction of infectious duration without symptoms. Using estimates from ref. 14, *ϕ* = 0.87. We chose *μ* = 0.015 d^−1^ to match the best estimates of infection fatality rate (= (1 – *ϕ*)*μ*/(*μ* + *γ*) ≈ 0.6%; ref. 15). Thus, *r*_max_ = 0.248 (0.17 to 0.23; ref. 5), *r*_min_ = −0.277, and *r̅* = −0.0145 d^−1^; *R*_*t*_ = *β*_*i*_/(*γ* + (1 – *ϕ*)*μ*) fluctuates between 1.77 (2.4 to 4; refs. 3, 5, 11) and 0.14. For *r̅* > 0, *β* varies between 0.67 and 0.12 d^−1^, respectively, with other parameters unchanged. Thus, *r*_max_ = 0.348, *r*_min_ = −0.202, and *r̅* = 0.0731 d^−1^; *R*_*t*_ fluctuates between 2.08 and 0.37.

### Simulating Dynamics.

For Figs. 1 and 2, we numerically solved Eq. **1** using ode45 in Matlab 2019b. Infection was initiated in population A at *t* = 0 with *I*_*A*_(0) = 10, and in population B at *t = τ* with *I*_*B*_(*τ*) =10, with no infection in B or movement between populations during 0 < *t* < *τ*. Growth rate at local initiation (*t* = 0, *τ* for A, B) is *r*_max_. Local transmission varies sinusoidally thereafter. We calculated cumulative cases after 50 cycles of Eq. **1**, ensuring disease fadeout. Global average initial growth rate is the asymptotic change in log total infections over a cycle after numerically solving Eq. **2** for 10 cycles. Code and other materials are at https://github.com/kortessis/SpatioTemporal_COVID-19.

## Data Availability

Code and other materials have been deposited in GitHub (https://github.com/kortessis/SpatioTemporal_COVID-19).
